# Viruses Like Sugars: How to Assess Glycan Involvement in Viral Attachment

**DOI:** 10.3390/microorganisms9061238

**Published:** 2021-06-07

**Authors:** Gregory Mathez, Valeria Cagno

**Affiliations:** Institute of Microbiology, Lausanne University Hospital, University of Lausanne, 1011 Lausanne, Switzerland; gregory.mathez@chuv.ch

**Keywords:** attachment receptor, viruses, glycan, sialic acid, heparan sulfate, HBGA, SARS-CoV-2, EV-D68

## Abstract

The first step of viral infection requires interaction with the host cell. Before finding the specific receptor that triggers entry, the majority of viruses interact with the glycocalyx. Identifying the carbohydrates that are specifically recognized by different viruses is important both for assessing the cellular tropism and for identifying new antiviral targets. Advances in the tools available for studying glycan–protein interactions have made it possible to identify them more rapidly; however, it is important to recognize the limitations of these methods in order to draw relevant conclusions. Here, we review different techniques: genetic screening, glycan arrays, enzymatic and pharmacological approaches, and surface plasmon resonance. We then detail the glycan interactions of enterovirus D68 and severe acute respiratory syndrome coronavirus 2 (SARS-CoV-2), highlighting the aspects that need further clarification.

## 1. Introduction

This review focuses on methods for assessing the involvement of carbohydrates in viral attachment and entry into the host cell. Viruses often bind to entry receptors that are not abundant on the cell surface; to increase their chances of finding them, they initially bind to attachment receptors comprising carbohydrates that are more widely expressed.

**Figure 1:**
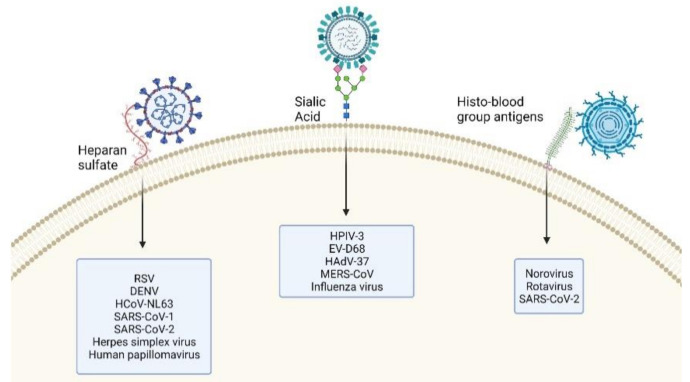
Glycosidic attachment receptor for viruses. Heparan sulfates, sialic acids and histo-blood group antigens are the main receptors used by the viruses to attach to the cell before infection. Examples of viruses binding to the different glycans are listed. Created with biorender.com.

The most common attachment receptors (Figure 1) are heparan sulfate (HS) proteoglycans, sialic acids (SA), and histo-blood group antigens (HBGA). HS are highly sulfated linear polysaccharides attached to a protein core [1]. They are involved in the cell attachment of many viruses including the herpes simplex virus, dengue virus (DENV), human papillomavirus, and respiratory syncytial virus (RSV) [1]. They have been reported to have a role in some coronaviruses, namely human coronavirus NL63 (HCoV-NL63) [2], severe acute respiratory syndrome coronavirus (SARS-CoV-1) [3], and SARS-CoV-2 [4]. They also have a role in the adaptation of clinical viral strains to in vitro culture [1,5].

SA are a group of 2-keto-3-deoxy-5-aminononulopyranosic sugars, the most common of which is N-acetyl neuraminic acid. They represent the terminal sugar on a wide variety of glycoproteins and glycolipids [6]. Due to their exposure on the cell surface and wide distribution, they play a role in numerous physiological and pathological functions, including the interaction with microorganisms [7,8,9]. They are best known for their involvement in the cell attachment of the influenza virus, but have been reported to have a role in numerous others including human parainfluenza virus type 3 (HPIV-3), human adenovirus type 37 (HAdV-37) [6], and Middle East respiratory syndrome coronavirus (MERS-CoV) [10]. The recognition between the SA and the viral glycoproteins is selective, with dependence on the linkage basis and SA modifications. For instance, SA with α2.3 linkage are recognized by avian influenza strains while SA with α2.6 linkage by human influenza strains [6].

HBGA, including the ABH antigens and Lewis antigens, are glycans expressed on blood cells, epithelial cells, and endothelial cells. They are involved in the viral binding of the norovirus [11], rotaviruses [12], and possibly SARS-CoV-2 [13].

Deciphering the role of glycans in the initial phases of the viral life cycle is fundamental to understanding viral tropism and adaptation. It also enables the identification of antivirals that target this step by mimicking or masking the glycans on the cell surface.

In this review, we discuss the different consolidated techniques available to identify the glycans necessary for viral infection. We then focus on those viruses for which the attachment receptors have been identified but further verification is needed, namely enterovirus D68 (EV-D68) and SARS-CoV-2.

## 2. Unbiased Methods: Unknown Carbohydrates

### 2.1. Glycomics

Different types of carbohydrate are present on cells and available for chemical synthesis or direct isolation from tissues. Probing the interactions of viral proteins or full viruses with a series of glycans of unknown specificity is an approach that has been widely used in recent years, both with glycan arrays and shotgun glycomics.

### 2.2. Glycan Arrays

Glycan arrays of both natural and synthetic origin are functionalized on glass slides and probed with the virus or viral glycoprotein of interest. Different types of conjugation have been explored, starting with biotinylated sugars on streptavidin plates, and successively replaced with N-hydroxysuccinimide- [14] or hydrazide- [15] activated glass slides.

Starting in 2001, the Consortium of Functional Glycomics (CFG) was among the first initiatives to develop this technology, providing arrays containing hundreds of glycans, interactive tools to evaluate binding, and open-access data. The preferences of several glycans have been analyzed with CFG arrays, including different strains of the influenza virus [16], human parainfluenza virus type 1 (HPIV-1) and HPIV-3 [17], and human adenovirus 3 (Ad3), Ad35, and Ad37 [18,19].

Currently, various arrays are available with differing functionalized glycans and characteristics both from private companies such as Z Biotech, Chemily, and RayBiotech [20] and from universities including Imperial College [20] and the Max Plank Institute [21].

Commercial products make it possible to probe the arrays directly under biosafety level (BSL)2, BSL3, or even BSL4 conditions. Studying the pathogens in their native form, without prior inactivation, does not alter their structure and preserves their glycan preference. However, one major limitation of these arrays is the absence of the full representation of the carbohydrates present at the site of infection, which has been partly overcome with shotgun glycomics.

### 2.3. Shotgun Glycomics

To assess the sugars present on a tissue of interest, the glycans on the array can be directly isolated from the tissue, separated with high-performance liquid chromatography (HPLC) and then printed onto the glass slide. After this step, hybridization will occur as described earlier. Once the carbohydrates bound by the virus are selected, their structures can be identified through mass spectrometry. This approach overcomes the limitation of identifying interactions with traditional arrays in which the glycans are synthetic and might not be present at the natural site of infection of the virus. However, due to the complexity of these techniques, the types of glycan printed might be affected by the treatment used to isolate, immobilize, and identify the sugars. This approach has been used to identify sugars bound by the influenza virus in porcine lungs [22] and decoy receptors for rotavirus present in human milk [23,24]. The first of these studies proved that influenza binds to N-linked glycans. By running the array from the porcine lungs and the CFG array in parallel, it was possible to identify unique glycans, and the partly overlapping results validated the approach.

In the analysis of the milk glycome, the goal was to identify the interactors of the rotavirus glycoprotein VP8 belonging to three different strains (two human and one bovine). The glycans were extracted from milk from either a single donor [24] or from multiple donors [23]. The interaction between the glycans and the virus has preventive effects, since the former act as decoy receptors. The results of this analysis were also compared with CFG array data. Both evidenced a lack of interaction with sialylated glycans, which were shown to have a role using other methods, and identified glycan structures that had previously been unknown [23]. Notably, the strains showed different specificities, although there were some similarities in the internal sugars. One limitation of this study, however, was the use of VP8 domains, as the presence of full virions might reveal additional interactions.

Shotgun glycomics can be useful to study alterations of the glycome in pathological conditions and the subsequent changes to assess dynamic rather than static viral interactions. For instance, HS are shed during inflammatory processes, and microorganisms such as bacteria or the influenza virus can expose different sugars on the cell surface due to their neuraminidase activity.

### 2.4. Genetic Approach

An alternative approach to identify the glycans that are essential for viral infection focuses on determining the enzymes that are involved in their biosynthesis through their genetic inactivation. This approach started with random inactivation in specific cell lines, particularly Chinese hamster ovary (CHO) cells in which mutations in the enzymes involved in HS biosynthesis were selected, and continued with the development of new technologies. This was initially achieved by screening a human haploid cell line, HAP1, in which the genes were randomly inactivated by a retroviral gene trap, and subsequently used clustered regularly interspaced palindromic repeats (CRISPR)/CRISPR-associated protein 9 (Cas9) libraries, which allowed the application of a similar method to a larger number of cell lines.

### 2.5. CHO Cells Mutagenized

The epithelial cell line CHO-K1 from Chinese hamster ovaries was mutagenized with ethyl methane sulfonate. Successive rounds of selection through ^35^S incorporation identified cells deficient in the synthesis of HS [22]. The mutants that are most commonly used in viral research are CHO-pgsA-745 cells, which lack xyloyltransferase I [22] and are deficient in all glycosaminoglycans, and CHO-pgsD-677, which lack N-acetylglucosaminyltransferase and glucuronyltransferase activities [23], and are deficient in HS synthesis. These cell lines were used to show the dependency on HS of several viruses including herpes simplex virus 1 (HSV-1) [24], cytomegalovirus (CMV) [25], RSV [26] and DENV [27]. Similarly, CHO cell lines that are deficient in SA synthesis are available [28].

### 2.6. Haploid Screening

Haploid screening is an unbiased method using HAP1 cells derived from chronic myeloid leukemia that have mostly haploid chromosomes [25]. Random mutations are introduced on these cells using gene-trap retroviruses [25]. After infection with the viruses, cells that are resistant to infection are cultured and deep sequencing can be used to find which genes were affected by the initial mutation [26,27,28]. To confirm the results of this approach, selective knockout of HAP1 cells can be carried out [26,28,29].

This method allows the identification of the host genes that are needed for the viral infection. In previous works, summarized in Table 1, it has led to the recognition of essential genes involved in the synthesis of SA for EV-D68 [29], α-dystroglycan glycosylation for the Lassa mammarenavirus (LASV) [26], HS expression for the vaccinia and Rift Valley fever viruses [27,28], and even receptor identification for adeno-associated viruses [30].

Although this method can identify new hits, it has several limitations. As these cells are not fully haploid [25], the recognition of genes located in chromosome 8, such as ST3GAL1, which is a beta-galactoside alpha 2,3-sialyltransferase [31], can be hampered since the other copy of the chromosome can rescue the mutation. The method is also limited to HAP1 cells; therefore, if these cells are not permissive for the virus of interest, haploid screening cannot be performed.

### 2.7. CRISPR/Cas9 Libraries

As an alternative to haploid screening, genome-wide CRISPR Cas9 screening can be performed. This method has allowed the identification of the host genes involved in viral infection in mammalian cell lines [35]. It consists of the expression of Cas9 in the cells of interest and the delivery of a single guide-RNA by a lentiviral vector [35]. The library of guide-RNAs used target multiple genes across the human or mouse genome [35]. After selection with an antibiotic, such as puromycin, and expansion of knock-out cells, they are infected once or multiple times [35,36,37,38,39]. The genomic DNA of the population that is resistant to viral infection is sequenced by next-generation sequencing [35,36,39]. Single knock-out with CRISPR-Cas9 is used as a validation experiment [36,37,39,40].

This screening has identified genes involved in the following: the HS expression necessary for Schmallenberg virus (SBV), DENV, Zika virus (ZIKV), and Sindbis virus infection (SINV) [37,38,39,40]; SA transport and cell intrinsic immunity for the influenza H5N1 virus [36]; dolichol-phosphate mannose synthesis for DENV [40]; and endosome-lysosome acidification for ZIKV [37] (Table 2).

Although this method allows the study of genes in various cell types, such as human-induced pluripotent stem-cell neural progenitors [37] and HAP1 cells [40], the main limitation is the library itself. Indeed, this screening is limited to the range of the library of guide-RNAs and cannot target all of the host genes. None of these methods on their own can directly prove either the requirement for a specific glycan for the entry of the virus or the direct interaction between the virus and the glycan. Further characterization is therefore needed.

## 3. Confirmatory Methods: Verification of the Glycan–Virus Interaction

Once putative glycans have been identified, additional verification of the interaction with the virus and the involvement of the glycan in the viral attachment must be performed.

The methods used can be multiple and complementary, spanning from microscopy techniques [41] to in silico modeling [4,42,43]; however, here, we focus on the enzymatic, pharmacological, and structural approaches.

### 3.1. Enzymatic Approach

The enzymatic removal of specific sugars can be used to evaluate the dependence on glycans for viral attachment to the cell, or its prevention in the case of decoy receptors. This method is largely used for multiple classes of glycans and is also a physiological response of the body.

In human cells, the only active enzyme able to cleave HS is heparanase I, which is an endo-β-D-endoglycosidase. It has a role in the modification of the extracellular matrix and in the biogenesis of exosomes; however, in pathological conditions, it has been shown to play an important role in tumorigenesis and angiogenesis [47]. Some viruses have been reported to upregulate its expression for efficient viral release [48].

For research purposes, heparinases of bacterial origin, which are used by microorganisms to take nutrients from glycosaminoglycans, are exploited. In particular, those most commonly used are purified from *Pedobacter heparinus*, previously called *Flavobacterium heparinum* [49]. Different subtypes have been identified: heparanase I cleaves both heparin and HS by acting on α-glycosidase linkage between hexosamines and O-sulfated iduronic acid residues; heparanase II cleaves heparin, HS hexosamines, and uronic-acid residues; and heparanase III is more specific for HS, cleaving hexosamines and uronic-acid residues in low-sulfated chains [50].

Heparinases are largely used to assess the HS dependence of viruses. For instance, some strains of enterovirus A71 can have increased HS binding, and mutations leading to this phenotype can occur even intra-host [51]. Their different attachment specificities can be clarified in the presence or absence of heparinases, either for individual viruses or in competition experiments among different variants [51].

The most commonly used of these are heparinase I and III, in some cases in combination [52]. To assess their effect on viral attachment, and not after viral replication, viral binding experiments are performed in which the amount of bound virus is evaluated through the quantitative polymerase chain reaction after incubation at 4 °C, which allows viral binding but not internalization. This complicates the readout, since heparinase treatment often shows a significant decrease, but the amount of viral genome associated with the cells remains high. These results should be carefully evaluated and complemented with additional experiments to assess whether there is only a partial dependency on HS, or ineffective cutting efficiency of the enzymes.

A valuable method for verifying the heparinase activity is to include a control virus that is known to depend on HS in the cell line of interest [1] or to reveal the exposures of internal domains with specific antibodies, such as anti-Δ-HS F69-3G10, which is specific for neo-epitopes generated after digestion [51].

Similarly, SA is cut by specific neuraminidase enzymes, normally from bacterial origin. Neuraminidases can have different specificities for branched or unbranched SA and on the linkage between the neuraminic acid and galactose (α2.6, α2.3, α2.8, or α2.9); careful evaluation of the best enzyme to use is therefore necessary.

Examples of viruses whose dependence on SA has been confirmed through enzymatic removal are EV-D68 [53] and rotaviruses [9,54]. For the latter, the use of attachment receptors appears to be strain-specific, and differs between animal and human strains. Classification of the rotavirus strains is based on their ‘sialidase resistance’ [9,54].

The enzymatic removal of SA has been addressed using a therapeutic approach with a study of DAS181, which is a recombinant sialidase catalytic domain from *Actinomyces viscosus* fused with a cell-anchoring peptide [55]. The compound proved to be active in vitro and in several animal models against different strains of influenza A and B, and parainfluenza. It has also been tested in clinical trials [56,57].

Different enzymes can also be used to remove specific residues, such as fucoses with α-fucosidases, which have been employed to assess the norovirus interaction with lettuce carbohydrates [58]. It is also possible to inhibit the synthesis of HBGA with 2-F-peracetyl-fucose, which has been adopted to assess the role of rotavirus infection in gut enteroids [59].

### 3.2. Pharmacological Approach

Competition experiments between the virus and the glycans can be performed in the presence of soluble molecules mimicking the sugars of interest or masking them on the cell surface.

This method has been largely used both to verify the interaction between the virus and the glycan, and to develop new antiviral treatments.

The most common HS mimetic is heparin, due to its commercial availability. However, there is high variability among the heparins used in terms of size (low molecular weight heparin or unfractionated) and origin (from porcine intestinal mucosa, calf lung epithelium, or synthetic origin). Moreover, it is important to consider that heparin is more sulfated than HS. Therefore, if heparin is used as a surrogate for HS, it might lead to an overestimation of the effect in viral attachment, as well as the binding strength. For a more comprehensive analysis of molecules interfering with viral HS binding and their stage of development, we refer to a previous publication [1]. The weaker strength of binding of SA compared to HS makes a multivalent interaction necessary to mediate inhibition; therefore, polymers, dendrimers, and other molecules have been studied to achieve viral inhibition [60,61]. A similar pharmacological approach is also under investigation for HBGA-mimicking molecules targeting noroviruses [62].

The inhibition of the virus–glycan interaction can also be mediated by lectins [63] or molecules interacting directly with the glycan of interest on the cell surface. For example, the bovine or human protein lactoferrin has been largely used for this purpose [3,64,65].

Major limitations of the use of this class of antivirals are the reversible nature of the interaction between the glycan and the virus, and the widespread distribution of the glycans of interest in the human body. These limitations hamper the development of these materials in vivo, unless endowed with irreversible mechanisms [61,66,67] or used with a topical approach to increase the chances of interaction with the virus without dilution in body fluids [68]. Some HS mimetics [69] or HS interactors [70] are currently in clinical trials, but none have yet been approved as an antiviral. To the best of our knowledge, the only antiviral molecules in commerce mimicking glycans are oseltamivir and zanamivir [71]. The drugs are sialic acid analogues designed to interfere with the neuraminidase activity of influenza virus, blocking the exit of the virus from the host cell [71].

### 3.3. Structural Approaches

Several methods are available to assess the direct interaction between the viral glycoproteins and the glycans. A non-exhaustive list includes the following: surface plasmon resonance (SPR) [72], nuclear magnetic resonance (NMR) [73], induced circular dichroism; biolayer interferometry; high resolution X-ray crystallography, cryo-electron microscopy, and atomic force microscopy [74]. Here, we focus on SPR due to its widespread use.

SPR is based on the immobilization of a ligand of interest on a sensor chip composed of a glass slide covered with gold. The sensor chip is connected to a prism and the change of the refraction angle of the incident light on the prism allows the measurement of the interaction between the immobilized ligands and an analyte in a flow chamber. This method is capable of measuring the interaction between proteins and determining the association and dissociation constant between viruses and glycans. It has been used to assess the interaction of human immunodeficiency virus (HIV) [75] and HSV-2 with HS [76], and has also recently been used extensively for SARS-CoV-2 [77,78].

Variability in measurements of similar ligands with SPR is linked to different methods of immobilization on the sensor chip. Although more complex, the incorporation of the ligand into a membrane allows proper orientation of the molecule and gives more reliable results. An additional limitation is linked to the use of single glycoproteins or only the receptor-binding domains of the glycoproteins instead of full viruses.

## 4. Glycan–Virus Interactions

Here, we focus on EV-D68 and SARS-CoV-2 due to the large amount of data available on their glycan–virus interactions as determined using the approaches previously described. However, some uncertainties persist in the characterization of their interactions. We suggest that additional experiments are needed to verify their interactions under relevant experimental conditions.

### 4.1. EV-D68 Dependency on Sialic Acid

EV-D68 is a single-strand positive RNA virus belonging to the *Picornaviridae*. It was originally observed in children with respiratory illness in 1962 [79]. After the 2014 outbreak, it was associated with acute flaccid myelitis. Successive studies have shown that some strains can replicate in neural cells in contrast with the original ones [53,80,81]. The dependency of EV-D68 on SA is under investigation.

Haploid genetic screening with a laboratory strain, Fermon, which was originally isolated in 1962, identified the requirement for several proteins involved in SA synthesis in HAP1 cells [29]. These results have been confirmed with mutant cells that cannot express SA and suggest a preference for α2.6 SA, which is present in the human upper respiratory tract [29]. The preference for this SA has also been observed with a glycan array and immunofluorescence experiments in the lower respiratory tract of ferrets [82,83]. Neuraminidase treatment reduced the efficiency of infection with the Fermon strain in induced pluripotent stem-cell motor neurons, HEK293T, A172, HeLa, RD, N2A, and human lung embryonic fibroblasts [84,85,86,87]. Preincubation with SA analogues was also effective [85]. Overall, the data support the use of SA by the Fermon strain to attach to cells. However, the picture is more complicated for clinical strains: some are dependent on SA [29,82], while others are able to infect cells after neuraminidase treatment or to infect cells deficient in the expression of SA [29,84]. These strains are not clade-specific [29].

Further investigation is needed to identify the reasons of the different attachment receptor usage, and the possible additional glycans involved in viral binding. For instance, one of the SA-independent strains identified was shown to interact with glycosaminoglycans in cells deficient for SA synthesis [34]. This interaction promoted the displacement of the pocket factor and the de-stabilization of the viral capsid leading to viral entry [34].

An important factor to take into consideration is the role of the cell lines used, which do not represent the natural viral tropism and might also play a role in the adaptation of the clinical strains. Studies in organoids or air–liquid interface (ALI) cultures might be more conclusive. Moreover, future work should be carried out with larger glycan arrays and natural glycans present at the site of infection, as so far, this has been carried out with only six sialyloligosaccharides [82].

### 4.2. SARS-CoV-2 Dependency on Glycans

SARS-CoV-2 is the cause of the ongoing pandemic of coronavirus disease 2019 (COVID-19), which has so far caused millions of deaths worldwide. The infection is mainly mediated by respiratory droplets, and the virus has been reported to infect different cells of the respiratory tract, although its tropism is not restricted [88]. The main receptor necessary for SARS-CoV-2 infection is the angiotensin-converting enzyme 2 (ACE-2). The interaction of the spike protein of SARS-CoV-2 with ACE-2, together with a conformational change mediated by proteases, either at the cell surface or in the endosomes, leads to viral entry [89].

However, ACE-2 expression in the respiratory epithelium is not as high as in other anatomical sites; therefore, in addition to the role of ACE-2, different glycans have been proposed as attachment receptors. In particular, HBGA and HS have been identified as possible interactors [4,13,52], while a potential inhibitory role has been described for SA [52].

For HBGA, a correlation between type A and a higher risk of respiratory failure with SARS-CoV-2 has been initially identified through the analysis of hospitalized patients [90] and genome-wide association studies in two different cohorts of patients [91].

A paper by Wu et al. [13] has evaluated the mechanistic reason behind this increased susceptibility. The receptor-binding domain of the spike, which shows sequence similarity to some galectin domains, has been shown to interact with the A type 1 glycan expressed in respiratory epithelial cells. By contrast, the RBD does not interact with either the A type 2 glycan expressed on red blood cells or B or 0 glycans. Although promising, these studies need further validation with the full virus and in relevant in vitro models.

Several papers instead suggest an interaction between SARS-CoV-2 and HS. These results are partly in line with the dependency on HS of other coronaviruses, in particular HCoV-NL63 [2] and SARS-CoV-1 [3].

Studies have been conducted with glycan arrays [92], SPR [77,78,92], pseudoviruses [93], and enzymatic removal of HS [4]. Here, we focus on the most relevant, highlighting the discrepancies among some results, the limitations and possible future investigations.

Several molecules interacting with or mimicking HS have been reported to exert antiviral activities against SARS-CoV-2 by acting on this putative mechanism, including human or bovine lactoferrin [64,65], sulfonated nanoparticles and cyclodextrins [94], and heparin and heparin analogues [52,77,93,95]. However, the range of inhibitory doses is variable, as is the strength of interaction between the spike and the heparin measured by SPR analysis [92]. These discrepancies warrant further investigation to understand the role of this interaction in vivo. Some influence could be attributed to the SARS-CoV-2 isolates used and their eventual cell adaptation, or to the cell lines. For instance, Clausen et al. [4] reported a lower need for HS in VeroE6 cells due to the higher expression of ACE-2. Moreover, Chu et al. [52] showed that in Calu3 and CaCo2 cells treated with high concentrations of soluble HS or with heparinase, the amount of bound SARS-CoV-2 decreased. Nevertheless, this inhibitory effect does not abolish the infection, suggesting a complementary yet non-indispensable role.

SA has been reported to have a detrimental role, with increased infection in the absence of this glycan [52]. The authors proposed a mechanism linked to the prevention of optimal interaction between ACE-2, which is sialylated, and the spike protein. However, an alternative explanation is suggested by a different publication [96]. The enhancement of infection after the removal of SA might be mediated by the exposure of lactosamine, which was the top hit of a ‘reverse’ glycan array performed by the authors. In this case, the spike protein was immobilized on the plate, biotinylated glycans were added, and the interaction was assessed by an enzyme-linked immunosorbent assay (ELISA).

These results suggest the possible involvement of different glycans in SARS-CoV-2 attachment; however, a validation with the full virus has often been missing and CRISPR/Cas9 screening has not identified any glycan biosynthetic enzyme as being essential for the virus [97]. Moreover, previous studies on respiratory epithelia and respiratory viruses have evidenced a lack of expression of HS in the apical side of the respiratory epithelium [98] and different receptor usage than that in cell lines [99]. Therefore, further validation is needed in relevant models such as ALI or in vivo models, in which the efficacy of HS or type A 2 HBGA analogues can be assessed, as well as enzymatic treatments to remove the glycans of interest. To date, the only published study validating the results in human lung explants has shown a modest reduction of viral infection at 24 hpi [54].

## 5. Conclusions

This review described the consolidated methods used to identify the glycans that interact with viruses in the initial steps of infection. The recent development of tools to investigate this interaction is expanding this field, and we propose a pipeline to identify the glycans of viruses for which no evidence currently exists.

An initial unbiased approach includes using large glycan arrays with GAGs, SA, and HBGA, or genetic approaches such as CRISPR/Cas9 libraries to identify the enzymes involved in the specific glycan biosynthesis. After the identification of the hits, it is necessary to verify the glycan with other methods, such as the enzymatic removal of the sugar of interest, competition experiments with soluble sugar mimetics, or measurement of the direct interactions between the carbohydrate and the glycoprotein.

It is important to take into consideration the limitations of the different methods and the validation of the results in different clinical strains, as highlighted by the data for EV-D68, and in relevant in vitro and in vivo models, which has been a missing factor for most of the studies with SARS-CoV-2 so far.

These considerations are particularly important in the context of antiviral development. Glycan analogues often failed during the preclinical or clinical studies. It is therefore fundamental to focus on molecules with high chances of success in vivo, i.e., endowed with irreversible inhibitory activity or administered topically. Otherwise, the risk is to over-interpret the in vitro results, as occurred during the SARS-CoV-2 pandemic, with the misuse of drugs and failure of clinical trials.

## Figures and Tables

**Table 1:** Glycan biosynthetic pathways identified with haploid screening.

Virus	Pathway Identified	Factor Identified	Reference
Chikungunya Virus	heparan sulfate	EXT1, EXT2, EXTL3, FAM20B, B3GAT3	[32]
Encephalomyocarditis Virus	sialic acid	SLC35A1, CMAS	[33]
	heparan sulfate	B3GAT3, SLC35B2	
Enterovirus D68	sialic acid	GNE, NANS, CMAS, SLC35A1, SLC35A2, MGAT5, B4GALT1, ST3GAL4, ST6GAL1	[29]
	heparan sulfate	B3GAT3, FAM20B, B3GALT6, B4GALT7, UXS1, XYLT2, EXT1, EXT2, EXTL3, UGP2, UGDH, SLC35B2, NDST1	[34]
Lassa Virus	sialic acid	SLC35A1, CMAS, SLC35A2, GNE	[26]
	N-glycosylation	ALG8, MAN1B1, ALG6, ALG5, MAN1A1, MGAT1	
	α-dystroglycan glycosylation	LARGE, ISPD, FKTN, FKRP, POMT1, POMT2, DPM3, C3orf39all	
Rift Valley Fever Virus	heparan sulfate	XYLT2, B4GALT7, B3GALT6, B3GAT3, EXTL3, EXT1, EXT2, NDST1, UXS1, UGDH, SLC35B2, PTAR1	[28]
Vaccinia Virus	heparan sulfate	XYLT2, B4GALT7, B3GALT6, B3GAT3, EXTL3, EXT1, EXT2, HS2ST1, NDST1, UGDH, UXS1, SLC35B2, PTAR1	[27]

**Table 2:** Glycan biosynthetic pathways identified with CRISPR/Cas9 screening.

Virus	Pathway Identified	Factor Identified	Reference
Dengue Virus	heparan sulfate	EXTL3, EXT2, B4GALT7, B3GALT6, B3GAT3, PAPSS1, SLC35B2	[40]
	dolichol-phosphate mannose synthetase	DPM1, DPM3	
Enterovirus D68	sialic acid	ST3GAL4	[41]
Hepatitis A virus	sialic acid	SLC35A1, UGCG, ST3GAL5, GNE, CMAS	[42]
Influenza H1N1	sialic acid	B4GALNT2	[43]
		SLC35A1, SLC35A2	[44]
Influenza H5N1	sialic acid	GNE, CMAS, SLC35A1, SLC35A2, GANAB, ALG12, ALG3, DPM2, ALG5	[36]
	glycan modification	A4GALT, B3GAT1, B4GALNT4, CHSY1, PIGN, CSGALNACT2, HS3ST6	
Japanese encephalitis virus	heparan sulfate	EXT1, EXT2, GLCE, HS6ST1, B3GAT3, B4GALT7, XYLT7, EXTL3, SLC35B2, GAA	[45]
Reovirus	sialic acid	NANS, ST3GAL4, SLC35A1, CMAS	[46]
Schmallenberg Virus	heparan sulfate	SLC35B2	[39]
Sindbis Virus	heparan sulfate	SLC35B2, B4GALT7, EXT2, EXT1	[38]
Vesicular Stomatitis Virus	sialic acid	SLC35A1	[44]
Zika Virus	heparan sulfate	TM9SF2, EXTL3, EXT2, NDST1, SLC35B2, EXT1, B4GALT7, PAPSS1, B3GALT6, HS6ST1	[37]

## Data Availability

Not applicable.
